# Here, There, and Everywhere: The Wide Host Range and Geographic Distribution of Zoonotic Orthopoxviruses

**DOI:** 10.3390/v13010043

**Published:** 2020-12-30

**Authors:** Natalia Ingrid Oliveira Silva, Jaqueline Silva de Oliveira, Erna Geessien Kroon, Giliane de Souza Trindade, Betânia Paiva Drumond

**Affiliations:** Laboratório de Vírus, Departamento de Microbiologia, Instituto de Ciências Biológicas, Universidade Federal de Minas Gerais: Belo Horizonte, Minas Gerais 31270-901, Brazil; nios2402@ufmg.br (N.I.O.S.); jaquelinebmedica@hotmail.com (J.S.d.O.); ernagkroon@gmail.com (E.G.K.); giliane@icb.ufmg.br (G.d.S.T.)

**Keywords:** *Orthopoxvirus*, *Poxviridae*, zoonosis, *Monkeypox virus*, *Cowpox virus*, *Vaccinia virus*, host range, wild and domestic animals, emergent viruses, outbreak

## Abstract

The global emergence of zoonotic viruses, including poxviruses, poses one of the greatest threats to human and animal health. Forty years after the eradication of smallpox, emerging zoonotic orthopoxviruses, such as monkeypox, cowpox, and vaccinia viruses continue to infect humans as well as wild and domestic animals. Currently, the geographical distribution of poxviruses in a broad range of hosts worldwide raises concerns regarding the possibility of outbreaks or viral dissemination to new geographical regions. Here, we review the global host ranges and current epidemiological understanding of zoonotic orthopoxviruses while focusing on orthopoxviruses with epidemic potential, including monkeypox, cowpox, and vaccinia viruses.

## 1. Poxvirus and Emerging Diseases

Zoonotic diseases, defined as diseases or infections that are naturally transmissible from vertebrate animals to humans, represent a significant threat to global health [1]. Among the species recognized as pathogenic to humans, more than half originated in animals, and some have been characterized as emerging or re-emerging pathogens [2,3]. Most zoonotic pathogens originated in wild and domesticated mammalian hosts such as bats, rodents, and primates [4]. The analysis of global trends indicates that new zoonotic threats will continue to emerge at an accelerating rate, and are mainly associated with an growthing population, changes in land use, climate changes, increased intercontinental travel, and expanded trade networks [4,5]. 

Poxviruses are of great veterinary and human importance and infect numerous vertebrate and invertebrate animals, including humans. The *Poxviridae* family is divided into two subfamilies, namely: *Chordopoxvirinae*, which infect vertebrates, and *Entomopoxvirinae* (A–C), which infect invertebrates. The *Chordopoxvirinae* subfamily is further divided into 18 genera (*Avipoxvirus, Capripoxvirus, Centapoxvirus, Cervidpoxvirus, Crocodylidpoxvirus, Leporipoxvirus, Macropopoxvirus, Molluscipoxvirus, Mustelpoxvirus, Orthopoxvirus, Oryzopoxvirus, Parapoxvirus, Pteropopoxvirus, Salmonpoxvirus, Sciuripoxvirus, Suipoxvirus, Vespertilionpoxvirus*, and *Yatapoxvirus*), distinguishable by their serological reactions [6,7].

The family *Poxviridae* comprises large, brick-shaped or ovoid enveloped viruses containing a linear, double-stranded DNA genome approximately 200 kilobase pairs in length [7,8]. Poxviruses are among mankind’s longest and best-known viruses mainly because of their most feared and lethal representative, *Variola virus* (VARV), the causative agent of smallpox. Before its remarkable eradication in 1980, VARV represented a centuries-old threat to humans worldwide and killed approximately 300–500 million people during the 20th century [9]. The global eradication of smallpox marked the culmination of an intensive vaccination program and quarantine measures promoted by the World Health Organization (WHO) [8,10,11]. Although VARV was eradicated 40 years ago, many challenges regarding poxvirus infections persist, including the worrisome possibility of VARV reintroduction by accidental release, its use as a biological weapon, or the emergence and re-emergence of zoonotic orthopoxviruses worldwide [12,13].

**Figure 1:**
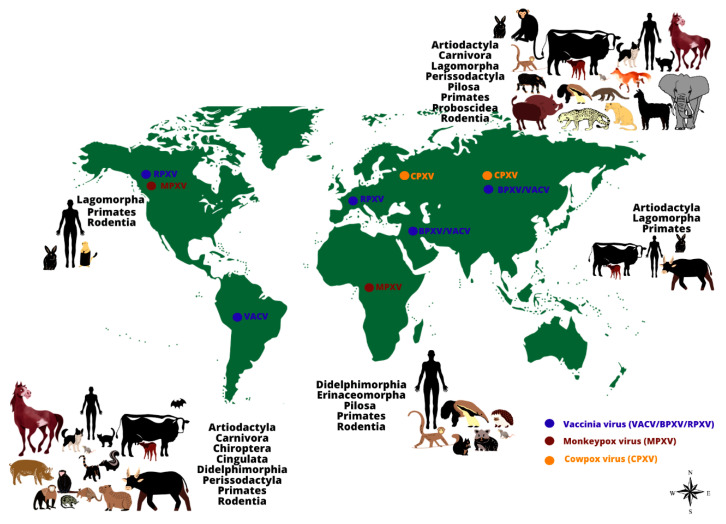
The worldwide distribution and host range of monkeypox, cowpox and vaccinia viruses.

Orthopoxviruses are remarkable for their wide host spectrum, ranging from humans to domestic and wild animals (Figure 1). *Orthopoxvirus* is the most important and well-characterized poxvirus genus, mainly due to its impact on human and animal health [7,8]. Here, we review the major aspects related to the dynamics and emergence of zoonotic orthopoxvirus infections worldwide, focusing on the host range and current epidemiological situation relating to monkeypox (MPXV), cowpox (CPXV), vaccinia (VACV), and VACV-like viruses.

The image shows the range of animal hosts (represented by orders) that have been demonstrated to be naturally infected by some *Orthopoxvirus* species, according to different regions of the world (except by Monkeypox virus in the United States of America, represented by imported cases). Orthopoxvirus infections have been demonstrated in animals belonging to different orders, using different methods (virus isolation, molecular detection of viral genomes or serological screening for antibodies against orthopoxviruses). The occurrence of some zoonotic orthopoxviruses has already been confirmed (by virus isolation or molecular detection of the viral genome) in some geographical regions (indicated by colored dots: blue: vaccinia virus (including buffalopox and rabbitpox viruses) in South America, Europe, Asia, and the Middle East; brown: monkeypox virus in Africa and North America; orange: cowpox virus in Europe and Asia).

## 2. Orthopoxvirus

The *Orthopoxvirus* genus comprises VARV, VACV, CPXV, MPXV, *camelpox virus*, *Akhmeta virus*, and other species with zoonotic potential. All orthopoxviruses share significant DNA sequence similarity and are immunologically cross-reactive and cross-protective. Infection with any orthopoxvirus is considered to generate protection against exposure or re- exposure to any other member of the genus [14,15]. Orthopoxvirus species are named primarily according to the hosts from which they were first isolated and identified; however, the name does not necessarily represents its natural reservoir or complete host range [8,16,17,18,19]. Despite the large number of studies, little is known about the primary hosts and reservoirs of zoonotic orthopoxviruses in nature, or their transmission and maintenance cycles [20]. Regarding the host range, orthopoxviruses can be both highly specialized and host restricted or generalists with a broad host range. For instance, VARV is a highly specialized virus that infects only humans, whereas MPXV, CPXV, and VACV are examples of generalist zoonotic orthopoxviruses that can infect several mammalian host species and also spillover into humans [20].

The evolution of generalists pathogens requires the successful crossing of host transmission barriers [21]. These include geographical, ecological, and behavioral constraints that separate a virus from its possible recipient hosts; virus-host cell incompatibility, such as tissue tropism, differences in receptor binding, genome replication, production, and shedding of infectious particles; and host immunity evasion, which includes cellular barriers or responses that restrict the infection and/or evasion of a virus from the innate immune system of its host [22]. To overcome these barriers, orthopoxviruses have different biological features that can synergistically contribute to the transmission to, and exploitation of, a broad range of new hosts species as observed for CXPV, MPXV, and VACV. Orthopoxviruses can cause both local lesions on the skin and systemic infections, resulting in direct and indirect transmission routes. When accompanied by viral particle stability in the environment, this can increase the likelihood of potential hosts being exposed to the virus independently of direct contact with infected hosts. In addition, orthopoxviruses can infect a variety of mammalian cells in a manner that is mostly independent of species-specific receptors and have large genomes that carry the information essential for viral replication, thereby increasing the possibility of successful infection in a new cell/host. Although the double stranded DNA genomes of orthopoxviruses have low mutation rates when compared with other viruses, such as RNA viruses, orthopoxviruses possess a genetic arsenal comprising several immune-regulatory, virulence, and host range genes [20]. The variety of host-genes among poxviruses enables them to express different viral proteins with important roles in cell tropism, as well as in the modulation of host signaling pathways and immunomodulatory responses, thereby establishing optimal cellular conditions for viral replication [23]. Finally, many of the strategies employed by orthopoxviruses to evade host immune defenses target conserved elements of the immune system in different potential hosts [20]. Combined, these features altogether are crucial for virus-cell and virus-host interactions and can contribute to the success of viral replication and transmission.

Despite the eradication of smallpox, the possibility of its re-emergence or the emergence of other orthopoxviruses in human and animal populations is a relevant global health issue. As smallpox vaccination is no longer mandatory, most of the world’s population that is under 40 years of age lacks immunity against orthopoxviruses [24,25]. This scenario is highlighted by numerous reports in recent years of human diseases caused by zoonotic orthopoxviruses such as MPXV [26,27,28,29,30,31,32,33], CPXV [34,35,36,37,38,39,40,41], VACV-like [42,43,44,45,46,47,48,49], and Akhmeta virus [18]. To date, the circulation of orthopoxviruses among wild and domestic animals has been recorded in different regions of the world, including South America, Africa, Europe, the Middle East, and Asia [27,40,42,43,50,51,52,53,54,55,56,57]. These facts raise concerns regarding the host ranges and distribution of orthopoxviruses, as well as their potential to cause outbreaks in animals and human populations, thereby further impacting animal and public health.

### 2.1. Monkeypox Virus

Monkeypox virus isolates are subdivided into two clades, namely, the West African and the Congo Basin clades, based on genetic and phenotypic (virulence) differences [58]. Notably, several studies have indicated that the clinical signs are similar between infections caused by viruses from either clades [59]. The first observation of MPXV infection was reported in 1958 during an outbreak of pustular rash illness in cynomolgus macaques (*Macaca fascicularis*) arriving in Copenhagen, Denmark, from Singapore [60]. Despite being named after the first described host, non-human primates are accidental hosts for MPXV [61].

Further insights into the range of taxa susceptible to MPXV infection were obtained by laboratory studies and field surveys. MPXV infections have been reported in a broad range of rodents, such as mice (*Mus musculus*), rabbits (*Oryctolagus cuniculus*), hamsters, woodchucks (*Marmota monax*), jerboas (*Jaculus* sp.), and porcupines (*Atherurus africanus*) (Table 1). Similarly, based on methods such as viral isolation, molecular assay, or experimental infection, susceptibility to MPXV infection was reported in ant-eaters (*Myrmecophaga tridactyla*), black-tailed prairie dogs (*Cynomys ludovicianus*), southern opossums (*Didelphis marsupialis*), short-tailed opossums (*Monodelphis domestica*), African hedgehogs (*Atelerix* sp.)*,* and several non-human primate species. Additionally, serological surveys have implicated several African rodents, including giant pouched rats (*Cricetomys* spp.), African dormices (*Graphiurus* spp.), rope squirrels (*Funisciurus* spp.), and sun squirrels (*Heliosciurus* spp.) as primary orthopoxvirus hosts in Africa [61,62,63].

Among Old World non-human primates, cynomolgus monkeys (*Macaca fascicularis*), sooty mangabeys (*Cercocebus atys*), orangutans (*Pongo pygmaeus*), and chimpanzees (*Pan troglodytes*) can be infected with MPXV. Among New World non-human primates [60,64,65,66,67,68,69,70,71,72,73,74,75], the common marmosets (*Callithrix jacchus*) was shown to be susceptible to MPXV infection through intravenous inoculation [76] (Table 1).

In 2003, a MPXV outbreak occurred in the United States of America (USA). Human infection was associated with direct contact with ill pet prairie dogs that were kept near to infected exotic animals imported from Ghana, West Africa [77]. This episode, as well as the infection of rodents, heightened concerns regarding the introduction of MPXV into the Americas. Meanwhile, the susceptibility of several African rodents to MPXV raised worries about the transmission of the virus to humans, as these animals are sometimes kept as pets [78,79].

Although humans are also accidental hosts [61], MPXV became the most significant pathogenic zoonotic orthopoxvirus for humans since the eradication of smallpox, given its associated morbidity (systemic infection) and lethality. The first human MPXV infection was described in 1970 for a 9-month old child in the Democratic Republic of Congo who had presented smallpox-like skin eruptions [70,80]. Several other human cases were reported in the following years. From 1970 to 1999, the WHO reported at least 404 confirmed and approximately 500 suspected human cases of monkeypox in different African countries (Central African Republic, Cameroon, Nigeria, Côte d’Ivoire, Liberia, Sierra Leone, and Gabon), but mainly in the Democratic Republic of Congo [52,81,82]. From the 2000s, alongside outbreaks in the Democratic Republic of Congo, the Republic of Congo, and South Sudan, the first human cases outside the Africa continent were also described. During May and June of 2003, cases of people with febrile illness and skin eruptions were notified to the Wisconsin Division of Public Health, but no deaths were reported and no person-to-person transmission was proven [78]. The source of this outbreak was traced back to imported infected exotic animals from Ghana [52,62,78]. Fortunately, the multi-state episode of captive rodent infection in the USA was short-lived, and the transmission cycle in the country was broken [83].

Alarmingly, several outbreaks of monkeypox in humans have been reported in African regions in the last decade. In 2010, two confirmed and eight suspected cases were described in the Republic of Congo related to the migration of refugees, regional inter-ethnic conflicts, or autochthonous cases. No deaths were reported among the confirmed cases, although one individual with suspected infection died [84]. In the same year, two cases of MPXV infection associated with hunting and the consumption of wild rodent meat were reported in the Central African Republic, with no deaths [85]. Numerous suspected and confirmed cases were reported in the Democratic Republic of Congo, from 2010 to 2016 [86,87], and in Serra Leone in 2014 [88]. Several suspected and 12 confirmed cases, as well as three deaths were reported in different provinces in the Central African Republic (Mbomou, Basse-Kotto, and Haute-Kotto) [52,89,90]. In 2017, the Republic of Congo reported its largest MPXV outbreak (88 suspected and seven confirmed cases, with six deaths), which affected 18 villages in five districts. This outbreak presented risks of MPXV spreading to neighboring countries given the extent of population mobility and refugee presence in the region [30].

Some African regions have continuously reported human cases of MPXV infection in recent years (2017 to 2020), including the Central African Republican (27 confirmed cases and two deaths) [91,92], Nigeria (181 confirmed cases and seven deaths) [31,93], Sierra Leone (one confirmed case) [94], Liberia (two confirmed cases and two deaths) [95], Cameroon (one confirmed case) [96], and the Democratic Republic of Congo (numerous confirmed cases and 321 deaths) [33,97]. Recently (2018), three cases of monkeypox were reported in the United Kingdom. Two were of people who had traveled to Nigeria, while the third concerned a health care worker who had had contact with one infected patient. One of the patients who had traveled to Nigeria reported having contact with a person with a rash and the possible consumption of bushmeat, raising the possibility that this may have been a case of secondary or even tertiary human-to-human transmission. Meanwhile, the infection contracted by the British care worker confirms human-to-human MPXV transmission [96]. Other cases of MPXV infections outside of Africa were reported in Israel (2018) and Singapore (2019), for travelers who imported the disease from Nigeria [98,99].

The natural source of MPXV and its maintenance cycle in nature remains unknown as the virus has only been isolated twice in nature (wild animals): once from the rope squirrel (*Funisciurus anerythrus*), Zaire, in 1985 [62], and once from the sooty mangabey (*Cercocebus atys*), Côte d’Ivoire, in 2012 [100]. To date, naturally occurring MPXV infections remain confined to the forest regions of West and Central Africa [101,102]. Consequently, a higher proportion of human MPXV cases are reported in regions (mainly African villages) where humans and non-human primates live in close proximity. The consumption, hunting, and handling of meat derived from non-human primates, rodents, and other small mammals have also been associated with human cases of MPXV infection [71,85,86,103,104,105]. Close contact with rodents has also been implicated as a source of human infection [67,106].

Human cases of monkeypox have been increasing even though they may have been underreported. Notably, diagnostic capabilities in the affected countries are most often limited, while health care workers worldwide are generally not aware of monkeypox disease. A lack of understanding about monkeypox disease associated with factors such as the increasing encroachment of humans into wild habitats, the inter-continental travel of people from endemic areas to MPXV-free regions, and the importation of animals both as pets and for laboratory studies raises concern regarding MPXV emergence, surveillance, prevention, and control [15]. Additionally, vaccination against smallpox was ceased decades ago, resulting in an increasingly larger number of people that are vulnerable to infection by MPXV or other orthopoxviruses. Although some animal species have been described as being susceptible to MPXV infection, most of what is known about its pathogenesis and clinical characteristics is derived from descriptions of animals in captivity or laboratory facilities. As monkeypox is an emerging zoonotic disease with epidemic potential and much of its host range and maintenance cycle in nature remains obscure, advances are urgently needed to better understand natural cycle of MPXV.

### 2.2. Cowpox Virus

Edward Jenner was the first to document CPXV infection after observing local lesions on the teats of cows, which he called “*cow-pox*”. Then, in 1798, Jenner demonstrated the efficacy of “*true cow-pox*” scarification in inducing immunity against smallpox [8,107]. There were frequent reports of bovine cowpox cases until the early 1970s in Europe, with sporadic transmission to humans, mainly milkers, occurring via contact with infected cows [108]. However, the number of bovine cowpox cases decreased, while reports of “cowpox-like” infections in several animal species, such as cats and elephants, [109] increased. “Cowpox-like” infections were described in a broad range of captive and domestic animals like non-human primates [110,111,112], felines [108,111,113,114,115,116,117], dogs [118], rodents [39,50,111,119,120,121,122,123,124,125], foxes [126,127], rhinoceroses [15,114,128], tapirs [129], okapis [130], horses [131], anteaters [114], mongooses [129], stone martens [132], bearcats [133], and farmed llamas [134,135] (Table 2). The viruses responsible for these infections induced clinical signs similar to those of CPXV infection such as hemorrhagic pocks on the chorioallantoic membrane and A-type inclusions bodies, and were thus considered to be “true cowpox” [136,137]. Most of these animals are thought to be accidental hosts for CPXV, and not reservoirs. Rodents, particularly voles (*Microtus* spp. and *Myodes* spp.), are known to be the primary CPXV reservoirs in nature [136,138].

CPXV is currently mostly found in Europe and northern and central Asia where cases of infections in rodents, cats, and humans continue to be reported. In Great Britain, CPXV is endemic in rodents such as bank voles (*Myodes glareolus*) and wood mice (*Apodemus sylvaticus*), while in Turkmenistan and Russia CPXV was isolated in the laboratory as well as in wild rodents [119]. Furthermore, serological surveys have also detected orthopoxvirus infections in France, Austria, and Norway in voles and wood mice [119]. Antibodies against orthopoxviruses were also detected in red foxes (*Vulpes vulpes*) in Western Europe being possibly related to CPXV infection, halthough red foxes are also known to be susceptible to ectromelia virus [119,139]. These reports of CPXV infection have occurred alongside an increasing number of reported infections in different animal species, leading to the designation of CPXV as an emerging health threat [140]. The first reported case of CPXV in a domestic cat occurred in 1977 in the Netherlands, and the number of CPXV infections in cats has since increased. According to Essbauer and collaborators (2010), more than 400 cases of CPXV infections in domestic cats were described in Western Eurasia until 2004 [15]. In cats, CPXV infection causes multiple skin lesions on the head, neck, forelimb, paws, and eyes (conjunctivitis), and the appearance of vesicles in the oral cavity and tongue. In the most severe cases, the disease can be systemic, affecting inner organs (mainly the lungs), with fatal outcomes being mostly associated with secondary bacterial infection [141]. Cats are the most affected domestic animals, mainly due to their predatory behavior against rodents, which are the CPXV reservoir in domestic and peridomestic environments [15,141,142,143,144]. However, the exact prevalence of feline cowpox is uncertain. CPXV infections in cats are mostly observed after increases in the rat population density [15,144].

The infection of pet rats and domestic cats by CPXV brings a higher risk of exposure to humans in the domestic environment, but rural or wild areas may be important as the source of infection [36]. Cowpox in humans is mainly caused by contact with infected domestic cats or rodents (such as *Rattus norvegicus*) that are kept as pets [15,34,37,38,121]. Even though human cowpox cases are usually self-limiting and not lethal, most people are susceptible to the disease, particularly children who are more often in close contact with pet animals [37,121]. The zoonotic potential of CPXV and its capacity to cause infection in wild and domestic environments are well established; however, many aspects of its natural maintenance cycle remains unknown. Besides the domestic animals, CPXV has a vast range of hosts and the increase in the breeding and commercialization of exotic animals raises concerns among health authorities regarding the emergence of cowpox, including in new geographical regions. 

### 2.3. Vaccinia Virus and Related Viruses

Although VACV is the most extensively studied orthopoxvirus, its origin remains unknown [145]. *Vaccinia virus*, the prototype species of the *Orthopoxvirus* genus, is best known as the live attenuated virus used worldwide by the WHO in the smallpox vaccine [146,147,148]. Despite the successful use of VACV as a vaccine, several vaccine strain-dependent complications have been reported, including progressive vaccinia, eczema vaccinatum, vaccinia gangraenosum, and neurological complications [145,149]. During smallpox eradication campaigns, various VACV strains with different degrees of virulence were used. The highly attenuated and modified VACV Ankara is a well-stablished third generation smallpox vaccine [150,151]. For a long time, VACV vaccine strains were assumed to be incapable of establishing a natural cycle due to their attenuation in the laboratory. However, several VACVs have been isolated from different host species, and in different locations around the world [42,152,153,154]. Similarly, sub-lineages of VACV (as buffalopox virus (BPXV) and rabbitpox virus (RPXV)) have been consistently isolated in different countries and from a wide range of hosts [14,15,16,43,53,155].

In India, BPXV was first described in 1934, and was responsible for infections that mainly affected domestic buffaloes (*Bubalus bubalis*), but also cows and humans [155]. BPXV resembles VACV in terms of its properties (size, shape, structure, and physicochemical properties) [156], pathogenesis, and pathology. Phylogenetic analyses confirmed the monophyly of the BPXV and its likely origin from the VACV Lister vaccine [155,156,157,158,159]. Since its first description, outbreaks of BPXV have been reported in India, Pakistan, Nepal, Egypt, Bangladesh, Indonesia, Russia, and Italy [15,43,53,160,161,162].

Humans become infected with BPXV through close contact with infected animals and no human-to-human transmission has been reported to date. In 2004–2005, a nosocomial outbreak in humans occurred in Pakistan, and the source of infection was traced to buffalo fat used as a first-aid medication for covering skin burns. This unusual source of infection was indicative of indirect BPXV transmission [14,163]. Additionally, a variety of animal species, such guinea pigs, BALB and white Swiss mice, cows, buffalo calves, rabbits, and chickens have been experimentally demonstrated to be susceptible to BPXV. Nevertheless, the role of these species in BPXV transmission and maintenance in nature remains unknown [44,164] and requires clarification.

RPXV is another VACV described as affecting different animal species worldwide (Table 3). RPXV was first described between 1930–1933 after outbreaks in laboratory rabbits in the USA. Additional outbreaks were later reported in 1941 in the Netherlands, while several other cases were also reported in Europe and the USA [165,166]. To date, no human transmission has been described for RPXV [165,167].

Different VACV isolates also circulate in South American countries, including Uruguay, Argentina, Colombia, and Brazil [54,55,56,168]. In the last few decades, several outbreaks of VACV infection have occurred in Brazil where the disease caused by VACV is popularly known as “*bovine vaccinia*”, due to its association with dairy cattle [42,168]. Bovine vaccinia is characterized by vesiculopustular exanthematous disease in cattle, anddairy workers who have direct contact with infected animals [169,170,171].

Since the detection of VACV in rural areas in Southeast Brazil, in 1999, several Brazilian-VACV (Br-VACV) isolates have been identified in the country (Araçatuba virus, Belo Horizonte virus, Cantagalo virus, Carangola eye virus 1, Carangola eye virus 2, Guarani P1virus, Guarani P2 virus, Mariana virus, Passatempo virus, Pelotas 1 virus, Pelotas 2 virus, and Serro virus) [148,169,172,173,174,175]. One hypothesis for the origin of the Br-VACVs assumes that they are derived from the spillback of a vaccine strain to the sylvatic environment [154,172], while another postulates that they may represent natural genetically and phenotypically diverse VACV populations, circulating in an unknown natural reservoir [148,152,173]. In particular, the presence or absence of an 18 nucleotide sequence within gene *A56R* gene (viral hemagglutinin) was proposed to be a molecular marker that can separate Br-VACVs into two distinct clades (group 1 and group 2) [176,177,178]. The existence of at least two clades was further confirmed through genetic and evolutionary analyses, of Br-VACVs, causing infection or co-infections in diversity of hosts in Brazil. [47,48,54,55,56,153,169,174,175,179,180,181,182,183,184,185,186,187,188]. In addition to the genetic diversity, some studies have also shown distinct biological profiles between the two Br-VACV groups [189,190]. The biological implications of this diversity in the context of the epidemiology and clinical evolution of the disease in humans should be further investigated.

Initially, VACV outbreaks were described as affecting dairy cattle and humans in rural environments. Consequently, the epidemiology of bovine vaccine in Brazil is associated with economic losses resulting from compromised milking herds [42,171,191,192]. In Brazil, bovine vaccinia have been mainly reported in the Southeast (Minas Gerais State), which has the largest dairy cattle herds in the country [42,148]. Nevertheless, VACV circulation in Brazil has already been documented for all the regions, affecting farm animals other than cattle, as well as wild animals [42]. Consistent with its wide geographical occurrence in Brazil, VACV has been detected in different biomes and related fauna. VACV genomes and antibodies against orthopoxviruses have been detected in a broad range of animalsincluding non-human primates (*Sapajus apella* and *Alouatta caraya*) [193]; procynoides (*Didelphis aurita, Didelphis albiventris,* and, *Nasua nasua*) [188,194]; cingulates (*Euphractus sexcintus*) [185]; marsupials (*Didelphis* sp. and *Caluromys philander*) [153,194]; bats (*Molossus rufus* and *Eumops perotis*) [185]; and wild rodents (*Oligoryzomys nigripes*, *Oligoryzomys flavescens*, *Sooretamys angouya*, *Calomys* sp., *Akodon* sp., *Necromys lasiurus*, *Necromys squamipes*, *Trinomys setosu*, *Cerradomys subflavus*, *Mus musculus*, *Rattus rattus,* and *Hydrochoerus hydrochaeris*) [153,180,185,195,196]. Furthermore, VACV has been detected in diverse peridomestic and domestic animals, including buffaloes [183,197,198], horses, donkeys [174,181,182,195], pigs [195], cows [195], dogs [188], cats [179], and mice [184] (Table 2).

Although direct VACV transmission between wild and domestic animals and between wild animals and humans has not been documented to date, these possibilities cannot be excluded. Several studies have indicated that cattle have a role as amplifiers in the bovine vaccinia cycle and have also demonstrated that VACV excretion in feces may favor viral transmission and its maintenance in the environment [170,199,200,201]. Subsequently, it was proposed that other farm animals could also be implicated in the VACV transmission chain, although direct transmission to humans has yet to be documented. Lastly, wild rodents could be VACV reservoirs, while peridomestic rodents could act as the link for VACV spread between wild and rural environments, promoting the transmission among wild mammals and farm animals [183,184].

Although VACV is known to have a broad range of hosts, many aspects of its natural history remain unknown. Bovine vaccinia is mainly caused by contact with infected cattle and is associated with economic losses to the dairy industry in Asia and South America [42,43,45,53,148]; however, the epidemic potential of VACV is a reality. Although VACV infection is usually self-limiting and not lethal, the disease profile in immunocompromised individuals may be differentially affected, presenting with severe and generalized manifestation [202], similar to that observed for cowpox. As currently documented for VACV, until the 1970s, CPXV mainly infected cattle and milkers. However, when cattle were replaced by cats and other animals as the primary hosts of CPXV infection, the number of human cases of CPXV infection increased. Given the similarities with CPXV, its plausible that VACV could follow similar path. Although farm animals are important sources of infection, the commercialization and consumption of dairy products could be alternative routes of zoonotic VACV transmission. In addition, VACV circulation in domestic animals such as cats and dogs bring the risk of viral transmission to humans in the domestic environment. The urban emergence of VACV could be an important health burden due to the unpreparedness of healthcare professionals to correctly identify and handle emerging cases [203]. Moreover, VACV infection presents a high attack rate, and VACV emerging cases in an urban area, where agglomeration of people is more frequent, could favor transmission, and trigger a public health emergency [148,204].

## 3. What Is Next for Monkeypox, Cowpox, and Vaccinia Viruses?

The history of poxviruses and orthopoxviruses has frequently been related to human cultural behavior. The establishment of agricultural settlements is considered one of the factors that favored the emergence of smallpox approximately 10,000 years ago. Orthopoxviruses continue to emerge and re-emerge due to the increasing proximity of humans to wild and rural habitats. Following smallpox eradication, the global scenario is marked by a vast naïve human population and the wide circulation of different orthopoxviruses. These facts raise concerns on the possible epidemic potential of these viruses in animals and humans. In fact, zoonotic orthopoxviruses already represent an important issue for animal health and economics. An example is the case of VACV and BPXV that have been associated with significant economic losses resulting from dairy cattle and livestock infection in several Asian and South American countries [42,43,53,191,192].

Currently, MPXV is mostly observed in Africa; CPXV in Europe and Asia; BPXV in Asia and the Middle East; and VACV in South America [53,54,55,56,119,140,160,162,168,205]. Although the factors that restrict the geographic distribution of some zoonotic orthopoxviruses are still unknown, their distribution range has been increasing as MPXV has been exported to parts of the USA [77], United Kingdom [96], Israel [98], and Singapore [99]. Legal or illegal trade of animals or animal-derived products, migration of animal populations, and traveling and migration of people are some factors that can contribute to the geographic dissemination of orthopoxviruses on local or global scales. Indeed, animal trade leading to the MPXV importation into the USA illustrated how globalization can favor the spatial spread of viruses [77]. On a local scale, migration of refugees within Africa is another example related to MPXV dissemination [84].

Because orthopoxviruses such as VACV, CPXV, and MPXV have genetic and phenotypic traits that allow them to possess a variety of mammals hosts [23], one cannot exclude the possibility of the virus infecting susceptible hosts in new geographical areas. These viruses are more prevalent in certain animal species, such as VACV in cattle and BPXV in buffalos. Molecular and immunological factors may be associated with productive infections in these animals while ecological factors may be linked to transmission between individuals of the same species. On the other hand, to cross host barriers and infect a new host, a virus must be able to infect and replicate in the new host, evade the immune system, and be efficiently transmitted [22]. Regardless of the remote possibility of an orthopoxvirus infecting new hosts, the current host plasticity is already notable, especially for MPXV, CPXV, and VACV. In addition, the emergence of a virus in a new host does not necessarily require evolutionary changes (mutations, rearrangements, etc.). One example of this process is the canine distemper virus, which has a very wide host range in mammals and its emergence in these species appears to be limited primarily by contact [22]. 

Orthopoxvirus outbreaks are usually related to populations living in rural areas or small villages. However, factors such as a high population density, increased urbanization, agriculture activities, deforestation, approximation to wild habitats, and inter-continental travel of people from endemic to pox-free regions may introduce poxviruses into different zones including urban environments [3,206]. A primary concern related to infected animals in periurban and urbanized environments is associated with a possible increase in orthopoxvirus transmission in a naïve population and even human-to-human transmission.

The epidemic potential of a virus is related to several factors, including geographical distribution, route of transmission, pathogenicity, and host range, among others. The epidemic potential may be lower for orthopoxviruses than for other RNA viruses or viruses transmitted by airway routes. Nevertheless, orthopoxviruses are remarkable regarding their transmission and dissemination among several hosts and environments. Wild and domestic animals could act as intermediate hosts for the emergence or re-emergence of orthopoxviruses in the human population. For instance, CPXV is transmitted from wild to domestic animals and then to humans, MPXV can be transmitted directly from wild animals to humans, and VACV is transmitted from domestic animals to humans [15,42,85,86,104,144]. Zoonotic orthopoxviruses may be transmitted either directly or indirectly and new forms of viral transmission have been described, which is a concern for public health. Milk and dairy products might be a potential source of VACV exposure or transmission [191]. Even under a low transmission rate, human-to-human transmission has already been demonstrated for zoonotic orthopoxviruses (MPXV and VACV) [96,207]. These are significant findings that should be further evaluated and closely monitored.

The cessation of routine vaccination against smallpox decades ago has resulted in a large contingent of people that are susceptible to orthopoxvirus infections, which have high morbidity rates. Moreover, in immunosuppressed individuals, exposure to orthopoxvirus infection can result in severe forms of the disease, or even death [208]. To date, there have been no reports of fatalities resulting from CXPV or VACV infection; however, MPXV infection in humans can progress to a lethality of up to 10% [209]. These facts indicate that VACV, MPXV, and CPXV pose a potential threat not only for humans, but for animals in different regions of the world. Together, these factors highlight the need for continuous epidemiological surveillance and the need to better understand the natural cycles and evolution of orthopoxviruses, their host range and reservoirs, the burden of outbreaks and dissemination of orthopoxvirus-associated diseases. This information is crucial for the development and application of control measures such as sanitary barriers and public policies aimed at controlling these viruses.

## Figures and Tables

**Table 1:** Hosts and susceptible animals to monkeypox virus infection.

Order/Family	Species	Method of Investigation *	Association to Human Infection **
Primates/ *Hominidae*	Humans (*Homo sapiens*)	viral isolation	yes
	Orangutans (*Pongo pygmaeus*)	viral isolation	yes
	Chimpanzees (*Pan troglodytes*)	viral isolation	no
Primates/ *Cercopithecidae*	Sooty mangabeys (*Cercocebus atys*)	PCR/ viral isolation	no
	Cynomolgus monkeys (*Macaca fascicularis*)	viral isolation	yes
Primates/ *Callithrichidae*	White-tufted marmosets (*Callithrix jacchus*)	Lab. Infec.	no
Rodentia/*Chinchillidae*	Rabbits (Oryctolagus cuniculus)	Lab. Infec.	no
Rodentia/*Muridae*	Inbred mouses (*Mus musculus*)	Lab. Infec.	no
Rodentia/*Cricetidae*	hamsters	Lab. Infec.	no
Rodentia/*Nesomyidae*	Giant-pouched rats (*Cricetomys* sp.)	PCR/ viral isolation	no
Rodentia/*Gliridae*	*African dormices* (*Graphiurus* sp.)	PCR/ viral isolation	no
Rodentia/*Sciuridae*	*Rope squirrels* (*Funisciurus* sp.)	PCR/ viral isolation	yes
	*Black-tailed prairie dogs* (*Cynomys ludovicianus*)	PCR	yes
	*Woodchucks* (*Marmota monax*)	PCR/ viral isolation	no
Rodentia/ *Dipodidae*	*Jerboas* (*Jaculus* sp.)	PCR/ viral isolation	no
Rodentia/*Hystricidae*	Porcupines (*Atherurus africanus*)	PCR/ viral isolation	no
Pilosa/*Macroscelididae*	Ant-eaters (*Myrmecophaga tridactyla*)	viral Isolation	no
Didelphimorphia/ *Didelphidae*	Southern opossums (*Didelphis* *marsupialis*)	PCR/ viral isolation	no
	Shot-tailed opossums (*Monodelphis* *domestica*)	PCR/ viral isolation	no
Erinaceomorpha/ *Erinaceidae*	African hedgehogs (*Atelerix* sp.)	PCR/ viral isolation	no

* Method of investigation: viral infection demonstrated by molecular assay (PCR) or viral isolation using samples obtained from naturally infected animals; Lab. Infec.: MPXV infection susceptibility was observed during experimental studies in laboratory. ** Transmission to humans already reported in the literature.

**Table 2:** Hosts and susceptible animals to cowpox virus infection.

Order/Family	Species	Method of Investigation *	Association to Human Infection **
Primates/*Hominidae*	Humans (*Homo sapiens*)	virus isolation	no
Primates/*Callithrichidae*	White-tufted marmosets (*Callithrix jacchus*)	virus isolation	no
Primates/*Cercopithecidae*	Barbary macaques (*Macaca sylvanus*)	virus isolation	no
	Cynomolgus macaques (*Macaca fascicularis*)	Lab. Infec.	no
	Rhesus macaques (*Macaca mulata*)	Lab. Infec.	no
Carnivora/*Felidae*	Domestic cats (*Felis catus*)	virus isolation	yes
	Cheetahs (*Acinonyx jubatus*)	virus isolation	yes
	Lions (*Panthera leo*)	virus isolation	no
	Pumas (*Felis concolor*)	virus isolation	no
	Black panthers (*Panthera padus*)	virus isolation	no
	Jaguarundis (*Herpailurus yaguarondi*)	virus isolation	no
	Jaguares (*Felis onca*)	virus isolation	no
Carnivora/*Canidae*	Dogs (*Canis lupus familiaris*)	virus isolation	no
	Foxes (*Vulpes vulpes*)	Lab. Infec.	no
Carnivora/*Herpestidae*	Banded mongooses (*Mungos mungo*)	virus isolation	no
Carnivora/*Ailuridae*	Bearcats (*Aiulurus fulgens*)	virus isolation	no
Perissodactyla/ *Rhinocerotidae*	Black rhinoceros (*Diceros bicornis*)	virus isolation	no
	White rhinoceros (*Ceratotherium s. simum*)	virus isolation	no
Perissodactyla/*Equidae*	Horses (*Equus caballus*)	virus isolation	no
Artiodactyla/*Bovidae*	Cows (*Bos taurus*)	virus isolation	yes
Artiodactyla/*Giraffidae*	Okapis (*Okapia johnstoni*)	virus isolation	no
Artiodactyla/*Camelidae*	Lamas (*Lama glama* sp.)	virus isolation	no
Rodentia/*Arvicolidae*	Field voles (*Microtus agrestis.*)	virus isolation	no
Rodentia/*Muridae*	Brown rats (*Rattus norvegicus*)	virus isolation	yes
	Giant gerbils (*Rhombomys opimus*)	virus isolation	no
Rodentia/*Cricetidae*	Root voles (*Microtus oeconomus*)	virus isolation	no
Rodentia/*Caviidae*	Patagonian cavys (*Dolichotis patagonum*)	PCR	no
Rodentia/*Castoridae*	Beavers (*Castor fibor canadensis*)	virus isolation	no
Rodentia/*Sciuridae*	Ground squirrels (*Citellus fulvus*)	virus isolation	no
Proboscidea/ *Elephantidae*	Asian elephants (*Elephas maximus*)	virus isolation	yes
	African elephants (*Loxodonta africana*)	virus isolation	no

* Method of investigation: virus infection demonstrated by molecular assay (PCR) or viral isolation using samples obtained fromnaturally infected animals; Lab. Infec.: MPXV infection susceptibility was observed during experimental studies in laboratory. ** Transmission to humans already reported in the literature.

**Table 3:** Hosts and susceptible animals to vaccinia and vaccinia-like viruses infection.

Order/Family	Species	Method of Investigation *	Association to Human Infection **
Artiodactyla/*Bovidae*	domestic buffaloes (*Bubalus bubalis*)	PCR/ Viral isolation	yes
Artiodactyla/*Bovidae*	cattle/cows (*Bos taurus*)	PCR/ Viral isolation	yes
Primates/*Hominidae*	Humans (*Homo sapiens*)	PCR/ Viral isolation	yes
Primates/*Cebidae*	Capuchin monkeys (*Sapajus apella*)	PCR	no
Primates/*Atelidae*	Black-howler monkeys (*Alouatta caraya*)	PCR	no
Didelphimorphia/*Didelphidae*	Black-eared possums (*Didelphis aurita*)	PCR	no
	White-eared possums (*Didelphis albiventris*)	PCR	no
	Wooly-cuycas (*Caluromys philander*)	PCR	no
Carnivora/*Procyonidae*	Ring-tailed coatis (*Nasua nasua*)	PCR	no
Carnivora/*Felidae*	Domestic cats (*Felis catus*)	PCR	no
Carnivora/*Canidae*	Domestic dogs (*Canis familiaris*)	PCR	no
Cingulata/*Chlamyphoridae*	Armadillos (*Euphractus sexcintus*)	PCR	no
Perissodactyla/*Equidae*	Horses (*Equus ferus caballus*)	PCR/ Viral isolation	yes
	Donkeys (*Equus africanus* sp.)	PCR	yes
	Mules (*Equus mulus*)	PCR	yes
Chiroptera/*Molossidae*	Black-molossus bats (*Molossus rufus*)	PCR	no
	Broad-eared bats (*Eumops perotis*)	PCR	no
Lagomorpha/*Leporidae*	Rabbits ^+^	PCR	yes
Rodentia/*Cricetidae*	(*Oryzomys* spp.)	PCR/ Viral isolation	no
	Black-footed colilargos (*Oligoryzomys nigripes*)	PCR	no
	Yellow pygmy rice rats (*Oligoryzomys flavenscens*)	PCR	no
	Rat-headed rice rats (*Sooretamys angouya*)	PCR	no
	Vesper mouses (*Calomys* spp.)	PCR	no
	Grass mouses (*Akodon* spp.)	PCR	no
	Hairy-tailed Bolo Mouses (*Necromys Lasiurus*)	PCR	no
	Bush mouses (*Cerradomys subflavus*)	PCR	no
Rodentia/*Echimyidae*	Hairy Atlantic spiny rats (*Trinomys setosus*)	PCR	no
Rodentia/*Muridae*	Inbred-mouses (*Mus musculus*)	PCR/ Viral isolation	yes
	Black-mouses (*Rattus rattus*)	PCR	no
Rodentia/*Caviidae*	Capybaras (*Hydrochoerus hydrochaeris*)	PCR	no

* Method of investigation: viral infection demonstrated by molecular assay (PCR) or viral isolation using samples obtained from naturally infected animals; Lab. Infec.: VACV infection susceptibility was observed during experimental studies in laboratory. ** Transmission to humans already reported in the literature. ^+^ Human infection from occupational exposure to rabbit skins inoculated with VACV.
